# The Mito-Hormetic Mechanisms of Ozone in the Clearance of SARS-CoV2 and in the COVID-19 Therapy

**DOI:** 10.3390/biomedicines10092258

**Published:** 2022-09-12

**Authors:** Salvatore Chirumbolo, Angelica Varesi, Marianno Franzini, Luigi Valdenassi, Sergio Pandolfi, Umberto Tirelli, Ciro Esposito, Giovanni Ricevuti

**Affiliations:** 1 Department of Neurosciences, Biomedicine and Movement Sciences, University of Verona, 37129 Verona, Italy; 2 Department of Biology and Biotechnology, University of Pavia, 27100 Pavia, Italy; 3 Almo Collegio Borromeo, 27100 Pavia, Italy; 4 Italian Scientific Society of Oxygen Ozone Therapy (SIOOT), 24100 Bergamo, Italy; 5 Tirelli Clinical Group, 33170 Pordenone, Italy; 6 Unit of Nephrology and Dialysis, ICS Maugeri, University of Pavia, 27100 Pavia, Italy; 7 Department of Drug Sciences, University of Pavia, 27100 Pavia, Italy

**Keywords:** anti-inflammatory, anti-oxidant, COVID-19, PASC, post-COVID, ozone therapy, medical therapy, review

## Abstract

An increasing body of evidence in the literature is reporting the feasibility of using medical ozone as a possible alternative and adjuvant treatment for COVID-19 patients, significantly reducing hospitalization time, pro-inflammatory indicators, and coagulation markers and improving blood oxygenation parameters. In addition to the well-described ability of medical ozone in counteracting oxidative stress through the upregulation of the main anti-oxidant and scavenging enzymes, oxygen–ozone (O_2_–O_3_) therapy has also proved effective in reducing chronic inflammation and the occurrence of immune thrombosis, two key players involved in COVID-19 exacerbation and severity. As chronic inflammation and oxidative stress are also reported to be among the main drivers of the long sequelae of SARS-CoV2 infection, a rising number of studies is investigating the potential of O_2_–O_3_ therapy to reduce and/or prevent the wide range of post-COVID (or PASC)-related disorders. This narrative review aims to describe the molecular mechanisms through which medical ozone acts, to summarize the clinical evidence on the use of O_2_–O_3_ therapy as an alternative and adjuvant COVID-19 treatment, and to discuss the emerging potential of this approach in the context of PASC symptoms, thus offering new insights into effective and safe nonantiviral therapies for the fighting of this devastating pandemic.

## 1. Introduction

Ozone, an allotrope of oxygen, made by three oxygen atoms, owes this odd name to the Greek word ὄζειν (smelling), attributed by Christian Friedrich Schönbein in 1840, due to its typically pungent odor. According to experimental evidence from microwave spectroscopy, ozone is a triatomic molecule, with a similar symmetry to the water molecule. The bond distances are 127.2 × 10^−12^ m, while the O-O-O angle is 116.78°, making it a polar molecule [1].

The molecule can be represented as a resonant hybrid with two boundary structures, each with a single bond on one side and a double bond on the other.

As an allotrope of oxygen, ozone is depicted as a kind of “super-oxygen” or “activated oxygen”, yet surprisingly ruling, when used in the medical therapy, a leading role in the complex response of the cell’s oxidative stress. Despite ozone being a “super oxygen”, it is not properly considered a super oxidant in medical therapy but is an anti-oxidant [2,3,4,5]. This paradoxical hallmark of ozone cannot come simply from its chemical nature but, on the contrary, by its ability to interact with highly complex, inter-related, and chaotic systems inside the biochemical machinery of signaling pathways and organelles’ turnover, which characterize the core life of a cell [6].

Briefly speaking, medical ozone is the pro-oxidant ozone used to trigger an anti-oxidant response. This may appear quite odd but there is no difference from the chemical ozone; the real discriminant is “how to use ozone” in its interplay with a biological system. A naïve, imaginative example may be how to use a bullet to get down or to move a jar with one swipe. The same toxicant is used, paradoxically, to earn benefits.

On the other hand, this Janus-like concept should also be applied to reactive oxygen species (ROS), which are well-known harmful substances, i.e., they should cause chemical damages to macromolecules [7,8] but are also less known signaling molecules, despite the controversial opinion having been raised as ROS are toxicants within the cell [9]. The fundamental tenet of a cell life is that very common molecules, widely ubiquitous in the organism, are used also as regulatory and tuning biomolecules. Therefore, it may not be so unusual that ROS are used as signaling elements [10,11], either in some pathologies with detrimental consequences [12] or in tissue remodeling, with rejuvenation outcomes [13].

The major playground where ROS should act as signaling molecules should be the complex mitochondria-endoplasmic reticulum (ER) or the recently described “mitochondria-associated endoplasmic reticulum membranes” or MAMs, previously suggested by one of us a few years ago [14,15]. MAMs regulate the fundamental items of a cell’s life, including its survival and response to stress, therefore regulating calcium transport and lipid synthesis, mitochondria biogenesis and dynamics, autophagy and apoptosis, and the activity of the inflammasome, suggesting, therefore, that MAM integrity is crucial for the cell [15].

How can ozone work in this system regarding COVID-19?

## 2. The Role of Ozone in the Mito-Hormetic Machinery

### 2.1. Insights on the Anti-Oxidant/Pro-Inflammatory Relationship Caused by Ozone

A possibility, though yet speculative thus far, is that ozone may target chloride channels in human lung epithelial cells and activate the Nrf2/Keap1/ARE system to buffer the possible damage from an impaired, exaggerated alteration of ozone-targeted chloride channels [16,17]. The activation of chloride channels modulation should inhibit the K^+^ efflux-dependent activation of the NLRP3 inflammasome, at the MAM level [15,18]. If confirmed, this mechanism should involve a subtle regulation of these participants in the final anti-inflammatory action of ozone, but, probably, ozone may act on multiple levels.

As a matter of fact, the inflammasome NLRP3 is activated by SARS-CoV2 in monocytes and macrophages [19], a circumstance that should be related to the ability of SARS-CoV2 to cause a dysfunctional impairment in mitochondria and their mitophagy mechanisms [20]. It is possible, therefore, to modulate the mitochondria-associated NLRP3 to activate the Nrf2 pathway, due to the well-known interplay between Nrf2 and inflammasomes [21]. This should be obtained by a biologically tuned and finely calibrated ozone medical administration via the many published reports on ozone therapy.

The possibility of a beneficial ozone directly from atmospheric ozone is excluded by the current scientific research, because of the recent observation that people living in near-coastal environments, notably rich in ozone and poor in engine-dust pollutants, were infected by SARS-CoV2 to a lesser extent than people living in continental, urban trafficking areas [22]. Probably, the anti-inflammatory role of ozone, by a direct action of this molecule, is mainly exerted by in situ innate immune cells [23,24].

The anti-oxidant and anti-inflammatory activity of medical ozone, i.e., gaseous ozone (usually in a balanced oxygen–ozone mixture), frequently injected as ozonated blood or administered as ozonate oil, relies on the ability of ozone, at relatively low dosages, to activate the cellular stress response via ROS signaling. This is globally known as “hormesis” and, as involving mitochondria, is also known as “mito-hormesis”, usually known to be considered in survival processes and antiaging [25,26].

SARS-CoV2 inhibits the expression of Nrf2-dependent genes, whereas Nrf2/Keap1/ARE agonists, such as 4-octyl-itaconate and dimethyl-fumarate, induce a strong anti-virus mechanism, able to prevent the SARS-CoV2 inhibiting effect on the Nrf2 pathway [27]. What we know is that the nuclear factor erythroid 2 (NFE2)-related factor 2 (Nrf2) is a molecule belonging to the cap ‘n’ collar (CNC) subfamily of basic region leucine zipper (bZip) transcription factors and mediates the gene expression of redox scavenging enzymes, such as glutathione S-transferase (GST) and NAD(P)H:quinone oxidoreductase 1 (NQO1), via the targeting of several antioxidants and electrophiles, including ozone-generated 4-hydroxynonenal (4-HNE) [28,29,30,31].

Gene expression requires a DNA sequence, known as the anti-oxidant response element (ARE), resembling the NFE-2 motif, thus giving Nrf2 the fundamental function of a xenobiotic-activated receptor (XAR), in order to detoxify cells from chemical stressors [32,33]. The first stressor-mediated activation of Nrf2, by ROS or ozone-derived electrophiles, such as 4-HNE, rapidly induces Nrf2 to modulate ROS production by mitochondria, therefore “adjusting” the proper level of ROS to lead them in acting as signaling molecules, rather than noxious toxicants [34,35,36,37].

The activation of the Nrf2 pathway, which is dampened by SARS-CoV2, regulates the fundamental survival function in cells targeted by the human coronavirus, as Nrf2 activation re-modulates the macrophage response in the innate immunity, by promoting mitochondrial fusion, hijacking the interferon response, and reprogramming mitochondria biogenesis and innate immunity [38].

As a biomodulator of the Nrf2-Keap1-ARE system, ozone should act as a functional bioregulator of the innate immune response to SARS-CoV2 [35].

The numerous papers describing the ability of oxygen–ozone to counteract SARS-CoV2 infections in progressing to more severe forms of COVID-19 [39,40,41,42,43,44,45] suggest that ozone is able to treat COVID-19 [46] and that the mechanism by which ozone targets SARS-CoV2 infection is of a hormetic nature [46] (Table 1).

Ozone does not actually directly elicit a pro-inflammatory response, in order to help innate immune cells in the virus clearance. Mitochondria regulates the activation of the nucleotide-binding oligomerization domain-like receptor family, pyrin domain-containing-3 (NLRP3) inflammasome [58]. Upon stress or cell microbial infection, mitochondria initiate the activation of the NLRP3 platform, leading to the caspase-1-mediated production of pro-inflammatory cytokines IL-1β and IL-18 [58]. According to Patergnani et al., mito-hormesis is the modulatory machinery on which particular molecules, known to have anti-oxidant properties, such as MitoQ, SkQ1, Szeto-Schiller 31 peptide, are able to modulate mitochondria dysfunction following a stressful input [59]. In this sense, as detailed further on, ozone should be able to finely adjust the mitochondria ability in managing a ROS-mediated signaling system, by mitohormesis.

Some fundamental proteins participate in the mechanism by which ROS act as signaling molecules rather than noxious oxidative and reactive species (hormesis), and one of the most investigated is thioredoxin-2 (Trx2) [60], a small protein with redox properties and double Cys-redox reactive sites (C90 and C93). The role of Trx2 is to maintain the reduced state of a cell, by oxidizing Cys disulfide bonds in a reversible way, and, in this sense, it regulates fundamental pathways via the apoptosis related-regulating kinase 1 (ASK-1) and the nuclear factor kappa B (NF-κB) [46]. The activation of Nrf2 by ozone-released mediators in the blood [35] activates the Trx and glutathione (GSH/GSSG) systems, thus inhibiting the apoptosis signals, via caspase 3 and 9 due to Trx2 depletion, and attenuates the TNF-mediated elevation of mitochondria ROS (mtROS) [60]. Briefly speaking, the activation of mitochondria-mediated inflammatory responses, to fight against SARS-CoV2, by mito-hormetic mechanisms, allows mitochondria to be safeguarded from mitophagy and dysfunction, and this mechanism can be held only by the activity of pro-oxidant molecules, such as ozone and its intermediates, in mito-hormetic dosages.

### 2.2. Insights into the Mito-Hormetic Ability of Ozone and Its Major Biochemical Mediators in Counteracting SARS-CoV2 Infection and COVID-19 Development: Endothelia and NO

The COVID-19 etiopathogenesis may be caused by impairment in the endothelia function of micro-circulation [35,61]. In this context, it is paramount to elucidate the role of NO, within the hormetic tenet, in the physiology of endothelia and the endothelia-platelet-immune interplay, particularly regarding the SARS-CoV2 infection.

The ability of mitochondria to maintain their chaotic dynamics, oscillating between fission and fusion, is highly regulated by nitric oxide (NO), as low concentrations of NO induce the mitochondrial biogenesis [62]. This mechanism is mediated by the activation of the cGMP-mediated pathway, related to an enhanced expression of PGC-1α, Tfam, and Nrf-1 [62], activating the mitochondria fusion [63]. The modest or slight activation of ROS, therefore, might be a key for triggering NO as a mediator in the inter-organelles, e.g., mitochondria and endoplasmic reticulum (ER) communication [63]. Actually, the endothelia nitric oxide synthase (eNOS) is the leading starter of NO able to induce mitochondria biogenesis; therefore, a cross-related interplay between endothelia and mitochondria from innate immune cells might be the major biochemical route where the mitohormetic activity of ozone and its mediators act as an inhibitor of SARS-CoV2 pathogenesis [64,65].

The electrophile molecule 4-hydroxy-trans-nonenal (4-HNE) is a well-known endothelia relaxing compound [66,67]. As many lipoperoxides (LOPs), which exert a hormetic activity [68], 4-HNE induces a mild NO production by regulating the activity of nitric oxide synthases [69]. The ozone-mediated production of ROS induces the formation of circulating lipoperoxides, and, in particular, the group derived from polyunsaturated fatty acids (PUFAs) can generate numerous α,β-unsaturated reactive 4-hydroxyalkenals, usually forming biochemical adducts with other macromolecules and including, besides 4-hydroxy-2E-nonenal (4-HNE), the compounds 4-hydroxyl-2E-hexenal (4-HHE) and 4-hydroxy-2E,6Z-dodecadienal (4-HDDE) [69].

All these LOPs mediate the activity of ozone from a direct toxicant compound to a hormetic-eliciting medical pro-drug. The ability to use a hormetic range of activity, notoriously given by adjusting a narrow interval of ozone dosages in the medical oxygen–ozone mixture, gives ozone the possibility to cause beneficial actions toward the therapy of COVID-19.

Experiments in RAW 264.7 macrophage cell lines showed that low doses of 4-HNE (0–100 μM, i.e., 0–15.6 μg/mL of 4-HNE) reduced the PMA-inflammatory activation of these cells, whereas higher doses of the LOP (200–300 μM, i.e., about 30–45 μg/mL of 4-HNE) increased the deleterious effect of PMA [70].

To determine whether the medical concentration of ozone, namely 30–85 μg/mL [46], should elicit a hormetic concentration of 4-HNE or other related LOPs, one should know that ozone is about tenfold more soluble with oxygen in blood plasmatic water [71]. If a usual medical ozone concentration, for example, 40 μg/mL (≈0.84 μmol/mL), is used, at least 78% of amino acids, within the first five minutes of ozone activity, may be oxidized, forming dehydro-L-ascorbate, and 20% of uric acid may be oxidized into allantoin [71,72] and only 10% of α-tocopherol may be oxidized into an α-tocopheryl radical [71]. Hence, a very modest amount of ozone should react with lipids generating LOPs, i.e., about 0.5–1.0% (i.e., 31–62 nM 4-HNE), therefore within the hormetic range [71].

The nitrite levels, in an in vitro study with RAW 264.7 macrophages, were maintained around 12–13 μmoles/L in the treatment of cells over 24 h with 1 to 100 nM 4-HNE [73]. The molecule 4-HNE leads to the circuital interplay between NO and Nrf2 in innate immune cells such as macrophages, by modulating the correct amount of NO acting on iNOS expression and in dependence of Nrf2 [73].

In conclusion, the role of 4-HNE in the therapy struggle against SARS-CoV2 may be outlined as a fine mechanism to trigger the interplay between Nrf2 and NO in the pro-inflammatory response against the viral entry and, at the same time, to regulate the role of endothelia by the NO/eNOS system.

Though somewhat speculative, this mechanism should elucidate how 4-HNE and other LOPs are able to elicit a pro-inflammatory response to SARS-CoV2 [46] without generating an alarming oxidative stress response with endothelia damage, a thromboembolic immune disorder, and or a patient’s exacerbation.

A possible reason is tentatively described in the next paragraph.

### 2.3. Insights into the Mito-Hormetic Ability of Ozone and Its Major Biochemical Mediators in Counteracting SARS-CoV2 Infection and COVID-19 Development: The HO-1 and the Hypoxic Pathway

Within the circulating blood, medical ozone also produces heme-oxygenase 1 (HO-1) [74], which is a fundamental regulator of optimal mitochondria functionality [75], a functionality that can generate hypoxia when mitochondrial function is impaired, in order to safeguard mitochondria survival [76]. Yet, hypoxia can be a fundamental factor of the severe progression of COVID-19 to ARDS [77].

During hypoxic conditions, macrophage HO-1 translocates into mitochondria, where it should activate ROS production and the autophagy markers LC3 and Drp-1 [78]; yet, mild doses of HO-1, inducing mito-hormesis [26], generates a short auto-loop circuit involving ROS (H_2_O_2_), Nrf2, and HIF-1α, which is an induced form of HO-1 [79], thus contributing to the fine regulation of mitochondria stability in a hypoxic micro-environment. The control on HIF-1α is mediated by NO, which, in turn, is counterbalanced by the activity of glutathione and peroxynitrite scavengers, so the global system NO-HIF-1α-HO-1-Nrf2 regulates the impact of the hypoxia on the cell viability via the mitochondria stability [80].

The hormetic activity of ozone might be simply restricted to the hypothesis that it assesses the mitochondria homeostasis, trying to reduce those pathways leading to an overwhelming of the oxidative stress threshold, where ROS activate a series of mechanisms leading to inflammation, autophagy, and/or apoptosis.

The activity of ozone on hypoxia has also been reported for the first and more represented cells that ozone meets during the oxygen ozone therapy, namely red blood cells [81]. The role of HIF-1α is crucial in the progress of SARS-CoV2 infection and should cause COVID-19 exacerbation in the case, reported above, of the oxidative stress response being overcome, associated with a sustained inflammatory response [82]. However, HO-1 is able to regulate HIF-1α, both in a downstream and upstream way, thus dampening the ischemic risk due to hypoxia [83]. In these circumstances, due to the stabilization of HIF-1α by HO-1 under critical illness conditions, the role of oxygen might be useless if HIF-1α uncouples mitochondria from oxidative respiration and induces the glycolytic pathway by inducing the expression of pyruvate dehydrogenase kinase 1 [84].

The hormetic activity on HIF-1α may be attained, probably, during a normoxic state, as HIF-1α is triggered by particular elements caused by an out-of-threshold level of oxidative stress response, including thrombin and activating the diacylglycerol-sensitive PKC and also NF-κB, including ROS, when activating the PI3K/p70S6K pathway and including transactivation at the Tyr-kinase receptors for HIF-1α [85]. In these circumstances, stabilization of HIF-1α may worsen the pathogenesis of COVID-19.

The normoxic control of HIF-1α stabilization, usually induced by angiotensin II, showed mechanisms very similar to the HIF-1α stabilization under hypoxic conditions, where H_2_O_2_ contributes to lowering the level of L-ascorbate (vitamin C) [85], a circumstance that might suggest a role of vitamin C in reducing COVID-19 exacerbation but with a complex and puzzling meaning.

The anti-viral activity of ozone and its lipoperoxide mediators should pertain, therefore, to very fine and complex mechanisms of auto-regulation, which involves the subtle balance between Nrf2 and NF-κB [35,86,87,88,89,90,91].

Further insights in the near future may elucidate this issue.

### 2.4. Ozone in COVID-19: The Key Mechanism of Ozone-Induced Hormetic Treatment against SARS-CoV2: Inflammasome, AhRs, and Nitric Oxide

**Figure 1:**
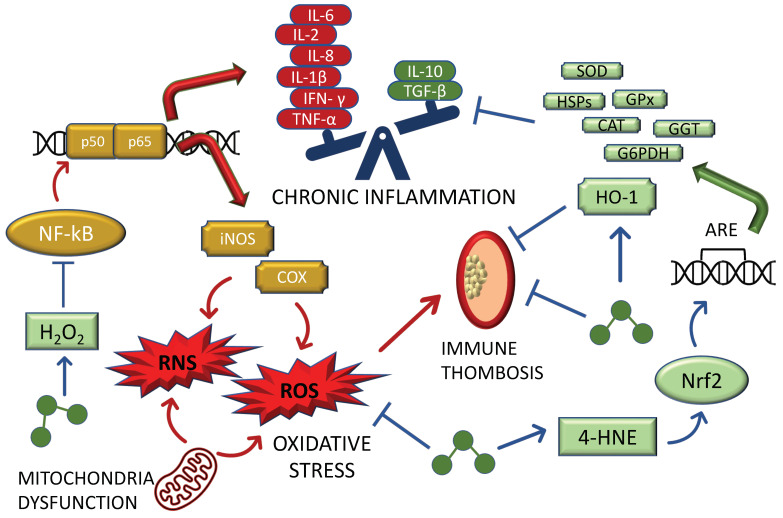
Medical ozone as alternative COVID-19 treatment: molecular insights. Following an increase in the expression of NF-kB, a situation of chronic inflammation characterized by an imbalance between pro-inflammatory cytokines and anti-inflammatory molecules is established. Concurrently, NF-kB-mediated signaling triggers the expression of iNOS and COX, which, in turn, promote the accumulation of ROS and RNS. Chronic inflammation and oxidative stress both lead to the formation of immune thrombosis, which are correlated with COVID-19 severity. Medical ozone can act both as an anti-inflammatory molecule (through the H_2_O_2_-mediated NF-kB inhibition) and as an anti-oxidant agent (through the 4-HNE-Nrf2-mediated transcription of various anti-oxidant effectors). Finally, the ozone-induced HO-1 elevation counteracts the formation of immune thrombosis. Ozone is represented by the picture with 3 green circles. Abbreviations: ARE: antioxidant response element; CAT: catalase; COX: cyclooxygenase; G6PDH: glucose-6-phosphate dehydrogenase; GGT: gamma-glutamyltransferase; GPx: glutathione peroxidase; HO-1: heme oxygenase 1; 4-HNE: 4-hydroxynonenal; HSPs: heat shock proteins; IFN-γ: interferon gamma; IL-10: interleukin 10; IL-1β: interleukin 1 beta; IL-2: interleukin 2; IL-6: interleukin 6; IL-8: interleukin 8; iNOS: nitric oxide synthase; NF-kB: nuclear factor kappa-light-chain enhancer of activated B cells; Nrf2: nuclear factor erythroid 2-related factor 2; RNS: reactive nitrogen species; ROS: reactive oxygen species; SOD: superoxide dismutase; TGF- β: transforming growth factor beta; TNF-α: tumor necrosis factor alpha.

The role of ozone in COVID-19 is multifaceted and deserves further insights, as thoroughly described in this manuscript (Figure 1).

As suggested in the Introduction, fundamentally, medical ozone does not act in humans by directly oxidizing molecular structures and inducing a strong oxidative response, because if so, ozone should generate a critical damage-mediated response and ultimately an injury. It is widely recognized that ozone in the blood, usually present in the oxygen–ozone autohemotherapy approaches or in ozonated whole-blood infiltrations, generates oxidized transient mediators, such as aldehydic electrophiles (for example, 4-hydroxynonenal) and lipoperoxides, collectively known as LOPs [35].

4-HNE is able to form chemical adducts with Cys residues, such as the Cys34 residue in albumin in picomolar concentrations (thus, in the hormetic range), adducts that trigger an oxidative stress response mediated by Nrf2 [92].

In the redox homeostasis, a panoply of molecules participates in the complex ROS-signaling-mediated response to stress. For example, the P450 cytochrome CYP1B1 is a key factor in the regulation of redox homeostasis [93]. During hyperoxic conditions, as occurring in COVID-19, CY1B1, whose production is enhanced by TNF-α, buffers ROS by forming oxidized adducts in DNA [94]; therefore, COVID-19 is a condition where the role of CYP1B1 as a key regulator of ROS as signaling molecules is impaired, due to TNF-α. Ozone can be “sensed” by cells via the aryl hydrocarbon receptors (AhRs) [95], and AhRs-downstream signaling activates the CYP1B1 [96]. It is well known that AhRs are targeted by hormetic-inducible anti-inflammatory molecules such as plant polyphenols and xenobiotics [97].

Recent evidence reported that the activity of 4-HNE is associated with a relevant up-regulation of AhRs [98]. The role of AhRs is much more intriguing than expected [99]. AhR is an exposome ligand, i.e., a transcriptional factor regulating numerous actions regarding plant-derived polyphenols, chemical toxicants and xenobiotics, endogenous byproducts, and microbiome catabolites [99]. Fundamentally, it is a detoxification factor. If ozone-derived ozonides, such as 4-HNE, 4-HHE, and PUFA-derived LOPs, target AhR, the translocase of outer mitochondrial membrane 20 (TOOM20) imports AhR into mitochondria [100]. LOPs activate AhRs, as well as 4-HNE [101], and most probably a slight induction of ROS enhances the rapid and significant expression of AhRs, thus preparing the cell to adjust the mitochondria-mediated response to stress, for example, eliciting a NLRP3-mediated response.

The way by which ozone is able to treat COVID-19 accounts for a wide panoply of biomolecular agents and signaling pathways.

The activation of the NLRP3 inflammasome, in the hormetic-AhR-mediated pathway, initiates a pro-inflammatory, anti-viral process, shut down by macrophage-released nitric oxide (NO) [102,103], which, in turn, inhibits the replication and life cycle of SARS-CoV2 in a dose-dependent manner [65]. In the presence of ROS, NO increases the lipid peroxidation, thus adding more LOPs signal to the activity of ozone [104]. These ozone-derived LOPs [35] target mitochondria, which even produce LOPs [105], therefore expanding the effect of ozone in the complex milieu of the oxidative stress/immune response to SARS-CoV2.

### 2.5. Ozone in Post-COVID-19 (PASC)

**Figure 2:**
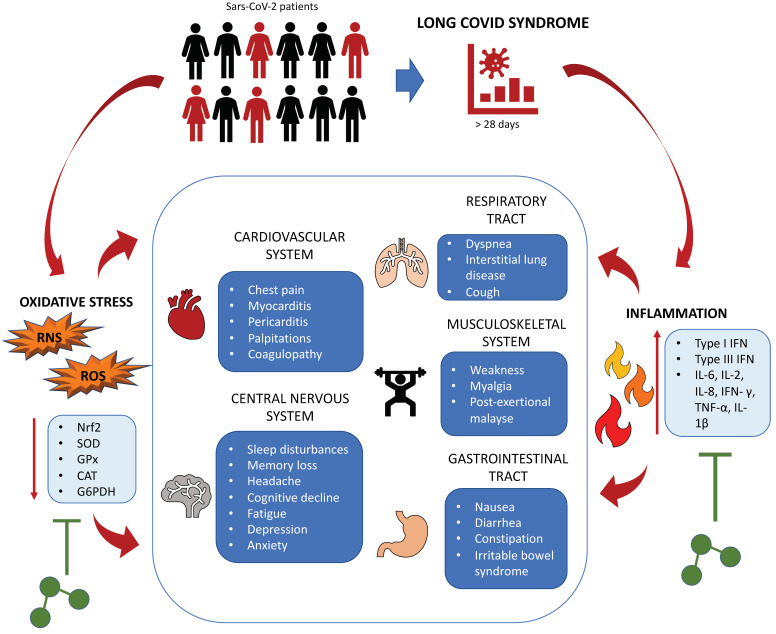
Possible therapeutic benefits of medical ozone in the treatment of PASC. PASC affects approximately one third of COVID-19 patients and is characterized by cardiovascular, respiratory, musculoskeletal, gastrointestinal, and brain dysfunctions. Oxidative stress and prolonged inflammation are thought to be responsible for the onset of these PASC-related disorders. The ability of ozone to re-establish the correct oxidant/antioxidant balance as well as its anti-inflammatory activity might be helpful in PASC treatment and/or prevention. Ozone is represented by the picture with 3 green circles. Abbreviations: CAT: catalase; G6PDH: glucose-6-phosphate dehydrogenase; GPx: glutathione peroxidase; IFN-γ: interferon gamma; IL-1β: interleukin 1 beta; IL-2: interleukin 2; IL-6: interleukin 6; IL-8: interleukin 8; RNS: reactive nitrogen species; PASC: post-acute sequelae of SARS-CoV2 infection; ROS: reactive oxygen species; SOD: superoxide dismutase; TNF-α: tumor necrosis factor alpha.

Post-COVID-19 syndrome, also known as post-acute sequelae of SARS CoV2 infection (PASC), is a long-term manifestation of the disease (≥28 days) reported in approximately one third of COVID-19 patients [106,107]. The typical PASC symptoms are not localized and include respiratory complications (i.e., dyspnea and interstitial lung disease), cardiovascular manifestations (i.e., chest pain, myocarditis, and pericarditis), musculoskeletal impairments (i.e., weakness and myalgia), cognitive damage (i.e., sleep disturbances, headache, and memory loss), gastrointestinal disturbances and systemic occurrences (i.e., fever, post-exertional malaise, and fatigue), among others [106,107,108,109] (Figure 2).

Yet, no effective therapeutic interventions exist for PASC patients to date.

In this context, although the use of O_2_–O_3_ therapy to prevent or resolve the PASC-related symptoms remain poorly investigated, some links between the supposed PASC pathophysiology and the benefits that ozone treatment may provide can be suggested. For example, it has been reported that innate immunity dysregulations characterize PASC patients [108,110,111]. Studies have shown that aberrant expression of type I and type III IFN, such as IFN-β and IFN-λ1, and an increased activity of some pro-inflammatory cytokines (i.e., IL-6 and TNF-α) are common molecular traits of PASC [108,112,113]. Of note, this prolonged inflammatory response was also found in PASC patients who contracted COVID-19 asymptomatically [114].

In this respect, there is evidence that O_2_–O_3_ therapy can counteract chronic inflammation by reducing the circulating levels of the main pro-inflammatory cytokines IL-6, IL-2, IL-8, IFN-γ, TNF-α, and IL-1β in a variety of disease contexts, including COVID-19 [6,35,115,116,117,118].

However, the role of O_2_–O_3_ therapy should be reported according to the ability of ozone in remodulating the T-reg response [119] and because, in PASC, mitochondria lose their membrane potential in immune cells [120]. Interestingly, 4-HNE is used as a prime target of Trx to recover the mitochondria membrane potential during an oxidative stress [121]. An interplay between 4-HNE and Trx occurs to re-establish the correct mitochondria membrane potential, following a viral insult [121].

The hormetic effect of ozone in PASC might be similar for many further pathologies with a complex immune dysregulation, loss of tolerance, and autoimmunity, where mitochondria functional impairment leads the scenery, for example, via IRGM1 [122]. Mitochondria dysfunctions join the two apparent opposite or concurrent events, inflammation and oxidative stress.

Due to the interplay between NF-kB and Nrf2 pathways, high levels of inflammation are often accompanied by elevated oxidative stress [35]. Together, inflammation and imbalance contribute to the onset of immune thrombosis, chronic inflammation, autoreactivity, fatigue, cognitive impairment, and pain, which are analogous to the classical PASC symptoms [123,124]. Moreover, it is known that viral replication is promoted in the presence of excessive pro-oxidant stimuli paralleled by the absence of sufficient antioxidant defenses, a condition that might favor the long-term persistence of the SARS-CoV2 genome found in PASC patients [40,125].

In this context, the ability of O_2_–O_3_ therapy to simultaneously act against inflammation and oxidative stress is of major interest. From a mechanistical point of view, O_3_ induces the formation of the second messengers H_2_O_2_, which acts as a fine-tuned modulator of NF-kB, and 4-HNE, a quite reactive aldehyde involved in the synthesis of antioxidant molecules and enzymes such as HO-1, SOD, CAT, GSH-Px, G6PDH, GSH, heat shock proteins, and γ-glutamyl transferase [6,126]. Moreover, activation of Nrf2 stimulated by O_3_ represses the activity of NF-kB together with the expression of its downstream players IL-6, IL-8, IFN-γ, TNF-α, cyclooxygenase-2 (COX-2), and inducible nitric oxide synthase (iNOS), thus reducing the levels of ROS and RNS, which are among the molecular mechanisms implicated in the systemic and neurological symptoms of PASC [5,127,128].

As a matter of fact, recent evidence has shown that certain polymorphisms in Nrf2 as well as in genes encoding for the antioxidant enzymes SOD2, GSTs, and GPx confer a high predisposition to the development of the neurological manifestations associated with PASC [129]. Considering these data, interventions capable of restoring the optimal oxidant–antioxidant balance should be considered not only in the context of SARS-CoV2 infection but also for the treatment of PASC [130], and O_2_–O_3_ therapy might be a leading candidate.

Rheumatologic and musculoskeletal disorders are among the main symptoms reported by PASC patients, with myalgia, joint pain, and fatigue being the most frequent [131,132,133]. As these disorders closely involve chronic inflammation and exaggerated immune activation, O_2_O_3_ therapy might interfere with the underlying pathophysiological processes, as already reported [134,135]. Other than acting on inflammation and oxidative stress [136], the benefits of ozone in reducing joint pain, back pain, knee osteoarthritis, and other musculoskeletal and rheumatoid conditions should also be ascribed to enhanced tissue oxygenation and improved protein and glucose metabolism [137]. Perhaps the most relevant results have been obtained using O_2_–O_3_ therapy to reduce the symptoms related to fibromyalgia and myalgic encephalomyelitis/chronic fatigue syndrome (ME/CFS), which closely resemble those reported by PASC patients. When O_2_–O_3_ therapy was administered to 200 ME/CFS patients, significant improvements in the fatigue rate were described in 77.5% of patients, with half of them reporting a total resolution of symptoms 30 days after treatment [138].

Similarly, 45 out of 65 patients diagnosed with fibromyalgia and receiving ozone by autohemotransfusion or rectal insufflation (as described in the SIOOT protocol) reported a significant amelioration of symptoms without showing any adverse effects [57].

Importantly, the same results have been reproduced on a cohort of 100 PASC patients, therefore confirming the great potential and safety of O_2_–O_3_ therapy in the treatment of chronic fatigue and systemic pain often found in individuals with PASC [139]. Although the same results show that females are more responsive than men, whether gender may influence the therapeutic outcome remains to be elucidated [35].

Chronic fatigue is often accompanied by a poor quality of sleep. In patients with insomnia and cardiovascular disease, low-dose ozone therapy substantially alleviated depression, anxiety, and sleep quality in a mechanism mediated by the increase in serum BDNF and GABA [140]. As the same symptomatology is shared with PASC, it would be interesting to see if similar results could be obtained in the context of PASC.

Strictly related to ME/CFS, neurocognitive difficulties often characterize PASC in a condition also called neuro-PASC [141]. Although not fully understood, the physiopathology behind these neurological manifestations might include viral invasion of the brain, excessive oxidative stress, neuroinflammation, and activation of glial cells [142]. In this respect, the encouraging results obtained in patients with cognitive frailty, neuroinflammation, and neurodegeneration [4,143,144] are at the basis of the possibility of using O_2_–O_3_ therapy to counter these symptoms, and studies on PASC patients are urgently required.

Concerning headaches, although long-term benefits have been reported by O_2_O_3_ autohemotherapy [145], improper ozone administration protocols may also induce encephalopathy and central nervous system toxicity [146], therefore underlining the double role of ozone based on dosages, methods, and timings of administration.

Lastly, an emerging player in the PASC pathophysiology is the dysbiosis of the microbiome [108]. As ozone therapy modulates the intestinal microbiota through the immunoregulatory effect exerted on circulating cytokines and T regulatory cells, its beneficial action can be extended to SARS-CoV2 infection as well as its post-acute sequelae [46].

Overall, the potential of O_2_–O_3_ therapy to limit and reduce the PASC symptoms is ensured by the multifunctionality of ozone, which can modulate a wide range of PASC manifestations. However, despite positive preliminary results, more evidence is needed to assess the feasibility, safety, and efficacy of O_2_–O_3_ therapy in the context of PASC.

## 3. Discussion

It is difficult to elucidate the actual role of ozone in the ability, by this oxygen allotrope, to counteract the SARS-CoV2 infection, either in the COVID-19 manifestation or in the PASC. Despite numerous investigators being prompted to trust the ability of ozone to induce a viral clearance by an inflammatory and immune response [46,147], the activity of ozone might be restricted to the ability of inducing toxic electrophiles and LOPs able to trigger ROS as signaling molecules. These compounds have a crucial role in the mitochondria and MAM bioactivity, in addition to mitochondria biogenesis [148,149].

Fundamentally, ozone does not necessarily enter the mitochondria and regulates their bioactivity in the production of ROS as signaling molecules. Aryl-hydrocarbon receptors should act as major actors in this mechanism. When linked to LOPs, AhRs are present also in mitochondria [100]. Probably, cytosolic proteins such as AIP and hsp90 should deliver a portion of the AhR to the internal mitochondrial space (IMS) via TOMM20, which is used to recognize proteins for the import of AhR into mitochondria.

Briefly speaking, this fine and highly regulated role of the import/export of AhR subunits from the cytosol to the mitochondria should finally regulate mitochondria survival, their biophysical and biochemical homeostasis, and, therefore, any complex signal regulating the cell stress response [100].

The way by which ozone acts even in a macroscopic way, involving macro-functions and organ functionality networks, has a fine, subtle key: the mitochondrion, with its complex relationship with other intracellular organelles and membranes. This is the keystone to comprehend the role of ozone therapy even in the SARS-CoV2 infection.

## 4. Conclusions

The ability of ozone, in the medical mixture oxygen–ozone, often used as autohemotherapy, rectal insufflation, or ozonized saline, to counteract the progression of SARS-CoV2 infection, lies in the ability of ozone to trigger the production of lipoperoxides, able to promote a mitohormetic response to the oxidative stress.

In this brief overview, we have described how many fundamental pathways and biochemical interplays ozone is able to elicit in order to activate a hormetic response to the oxidative stress, where the maintenance of the fine homeostasis of mitochondria stability and optimal function is crucial. In this context, ozone is able to polarize the ability of cells to counteract viral infection and COVID-19 pathology.

## Figures and Tables

**Table 1:** Clinical evidence for the use of medical ozone in COVID-19 treatment.

Ref.	Study Type	Number of Participants	Clinical Condition	Treatment	Results	Statistically Significant
[42]	RCT	60 patients (30 treated and 30 controls)	Mild to moderate COVID-19	Rectal insufflation + minor autohemotherapy	↓ CRP, LDH, ferritin ↑ SpO_2_	No
[44]	PCS	55 patients (37 treated and 18 controls)	Hospitalized	CT + major autohemotherapy (IV for seven consecutive days, 30 μg/mL)	↓ ICU hospitalization ↓ Mortality risk	No
[47]	CR	3 patients	Respiratory failure	Autohemotherapy (1–6 sessions)	↓ Hypoxia ↓ LDH, CRP, D-dimer ↓ Mechanical ventilation ↓ FiO_2_ ↑ PaO_2_	NA
[45]	CSS	50 patients	Hospitalized in ICUs presenting ARDS	4 cycles of autohemotherapy (SIOOT protocol), 30 μg/mL	↓ CRP, LDH, ALT, IL-6, D-dimer ↑ SatO_2_%	Yes
[43]	PCS	18 patients (9 treated and 9 controls)	Severe pneumonia	Autohemotherapy twice a day for 4 days (median), 40 μg/mL	↑ Recovery rate ↓ Time to reduce CRP, D-dimer, ferritin, LDH	Yes
[48]	CR	2 patients	Mild fever, dyspnea	Major autohemotherapy (daily for 7 days), 20 μg/mL	↓ CRP, LDH, IL-6 ↑ Recovery rate	NA
[49]	RCT	28 patients (14 treated and 14 controls)	Hospitalized with severe COVID-19	CT + autohemotherapy twice a day for 7 days	↓ Need for ventilation = Inflammatory markers = Lymphocyte subpopulations	No
[50]	CCS	28 patients (14 treated and 14 controls)	Bilateral COVID-19 pneumonia	Intra-rectal ozone daily for 8 days, 35 μg/mL	↓ D-dimer, fibrinogen, urea, CRP, LDH, IL-6, ferritin ↓ Radiological pneumonitis ↓ Leukocytes ↑ Lymphocytes ↓ O_2_ supply	Yes
[51]	CR	2 patients	Presenting hypoxia and requiring ventilation	Rectal ozone insufflation (1–2 sessions)	↑ SpO_2_	NA
[52]	RCCS	235 healthcare workers (64 treated and 171 controls)	Healthy	COVID-19 standard prophylaxis + IV ozonized saline daily for 4 days/month	↓ COVID-19 incidence	Yes
[53]	RCT	92 patients (48 treated and 44 controls)	Mild to moderate pneumonia	CT + autohemotherapy daily for 3 days, 40 μg/mL	↓ Leukocytes ↑ CRP = Mortality rate = Need for ventilation = Hospital stay	No
[54]	PCT	10 patients	Moderate COVID-19	CT + IV ozonized saline (200 mL daily for 8 days)	↓ CRP, D-dimer, IL-6 ↑ SpO_2_/FiO_2_	Yes
[55]	CCS	60 patients (30 treated and 30 controls)	Mild to moderate pneumonia	CT + autohemotherapy daily for 3 days, 40 μg/mL	↓ SIMEU clinical phenotype	Yes
[56]	RCT	30 patients (15 treated and 15 controls)		CT + inhalation of nebulized ozone, 3 sessions of 10 min (0.2 ppm/session) every 5 min daily for 5 days)	↓ Hospitalization time ↓ CRP = D-dimer, urea, LDH lymphocytes, leukocytes, platelets	Yes
[57]	Multicenter CSS	100 patients	PASC	Autohemotherapy (SIOOT protocol)	↓ Fatigue	Yes

Abbreviations: ARDS: acute respiratory distress syndrome; CCS: case-control study; CR: case report; CRP: C reactive protein; CSS: case series study; CT: conventional therapy for COVID-19; FiO_2_: fractional inspired oxygen; ICU: intensive care unit; IV: intravenous; LDH: lactate dehydrogenase; PaO_2_: arterial oxygen partial pressure in mmHg; PASC: post-acute sequelae of SARS-CoV2 infection; PCS: prospective, controlled study; PCT: pilot clinical trial; RCCS: retrospective controlled cohort study; RCT: randomized controlled trial; SatO_2_%: saturation of oxygen in percentage; SIMEU: Italian society of emergency-urgency medicine; SIOOT: scientific society of oxygen ozone therapy; SpO_2_: oxygen saturation; ↓: decrease; ↑: increase.

## References

[1] Ivanov S.V., Panchenko V.Y. (1994). Infrared and microwave spectroscopy of ozone: Historical aspects. Phys. -Uspekhi.

[2] Bocci V. (2006). Is it true that ozone is always toxic? The end of a dogma. Toxicol. Appl. Pharmacol..

[3] Bocci V., Borrelli E., Travagli V., Zanardi I. (2009). The ozone paradox: Ozone is a strong oxidant as well as a medical drug. Med. Res. Rev..

[4] Scassellati C., Galoforo A.C., Bonvicini C., Esposito C., Ricevuti G. (2020). Ozone: A natural bioactive molecule with antioxidant property as potential new strategy in aging and in neurodegenerative disorders. Ageing Res. Rev..

[5] Galiè M., Covi V., Tabaracci G., Malatesta M. (2019). The Role of Nrf2 in the Antioxidant Cellular Response to Medical Ozone Exposure. Int. J. Mol. Sci..

[6] Viebahn-Haensler R., Fernández O.L. (2021). Ozone in Medicine. The Low-Dose Ozone Concept and Its Basic Biochemical Mechanisms of Action in Chronic Inflammatory Diseases. Int. J. Mol. Sci..

[7] Ezraty B., Gennaris A., Barras F., Collet J.-F. (2017). Oxidative stress, protein damage and repair in bacteria. Nat. Rev. Microbiol..

[8] Juan C., de la Lastra J.P., Plou F., Pérez-Lebeña E. (2021). The Chemistry of Reactive Oxygen Species (ROS) Revisited: Outlining Their Role in Biological Macromolecules (DNA, Lipids and Proteins) and Induced Pathologies. Int. J. Mol. Sci..

[9] D’Autréaux B., Toledano M.B. (2007). ROS as signalling molecules: Mechanisms that generate specificity in ROS homeostasis. Nat. Rev. Mol. Cell Biol..

[10] Mittler R., Vanderauwera S., Suzuki N., Miller G., Tognetti V.B., Vandepoele K., Gollery M., Shulaev V., Van Breusegem F. (2011). ROS signaling: The new wave?. Trends Plant Sci..

[11] Zhang J., Wang X., Vikash V., Ye Q., Wu D., Liu Y., Dong W. (2016). ROS and ROS-mediated cellular signaling. Oxid. Med. Cell. Longev..

[12] Phull A.-R., Nasir B., Haq I.U., Kim S.J. (2018). Oxidative stress, consequences and ROS mediated cellular signaling in rheumatoid arthritis. Chem. Interact..

[13] Rigotti G., Chirumbolo S. (2018). Biological Morphogenetic Surgery: A Minimally Invasive Procedure to Address Different Biological Mechanisms. Aesthetic Surg. J..

[14] Chirumbolo S., Bjørklund G. (2017). PERM Hypothesis: The Fundamental Machinery Able to Elucidate the Role of Xenobiotics and Hormesis in Cell Survival and Homeostasis. Int. J. Mol. Sci..

[15] Degechisa S.T., Dabi Y.T., Gizaw S.T. (2022). The mitochondrial associated endoplasmic reticulum membranes: A platform for the pathogenesis of inflammation-mediated metabolic diseases. Immun. Inflamm. Dis..

[16] Canella R., Martini M., Borriello R., Cavicchio C., Muresan X.M., Benedusi M., Cervellati F., Valacchi G. (2017). Modulation of Chloride Currents in Human Lung Epithelial Cells Exposed to Exogenous Oxidative Stress. J. Cell. Physiol..

[17] Canella R., Benedusi M., Martini M., Cervellati F., Cavicchio C., Valacchi G. (2017). Role of Nrf2 in preventing oxidative stress induced chloride current alteration in human lung cells. J. Cell. Physiol..

[18] Swanton T., Beswick J.A., Hammadi H., Morris L., Williams D., de Cesco S., El-Sharkawy L., Yu S., Green J., Davis J.B. (2020). Selective inhibition of the K+ efflux sensitive NLRP3 pathway by Cl− channel modulation. Chem. Sci..

[19] Kaivola J., Nyman T.A., Matikainen S. (2021). Inflammasomes and SARS-CoV-2 Infection. Viruses.

[20] Shang C., Liu Z., Zhu Y., Lu J., Ge C., Zhang C., Li N., Jin N., Li Y., Tian M. (2022). SARS-CoV-2 Causes Mitochondrial Dysfunction and Mitophagy Impairment. Front. Microbiol..

[21] Hennig P., Garstkiewicz M., Grossi S., Di Filippo M., French L.E., Beer H.-D. (2018). The Crosstalk between Nrf2 and Inflammasomes. Int. J. Mol. Sci..

[22] Chirumbolo S., Bjørklund G. (2020). The bimodal SARS-CoV-2 outbreak in Italy as an effect of environmental and allergic causes. J. Allergy Clin. Immunol..

[23] Kettle A.J., Winterbourn C.C. (2005). Do neutrophils produce ozone? An appraisal of current evidence. BioFactors.

[24] Babior B.M., Takeuchi C., Ruedi J., Gutierrez A., Wentworth P. (2003). Investigating antibody-catalyzed ozone generation by human neutrophils. Proc. Natl. Acad. Sci. USA.

[25] Bárcena C., Mayoral P., Quirós P.M. (2018). Mitohormesis, an Antiaging Paradigm. Int. Rev. Cell Mol. Biol..

[26] Ristow M., Schmeisser K. (2014). Mitohormesis: Promoting Health and Lifespan by Increased Levels of Reactive Oxygen Species (ROS). Dose-Response.

[27] Olagnier D., Farahani E., Thyrsted J., Blay-Cadanet J., Herengt A., Idorn M., Hait A., Hernaez B., Knudsen A., Iversen M.B. (2020). Author Correction: SARS-CoV2-mediated suppression of NRF2-signaling reveals potent antiviral and anti-inflammatory activity of 4-octyl-itaconate and dimethyl fumarate. Nat. Commun..

[28] Ma Q. (2013). Role of Nrf2 in Oxidative Stress and Toxicity. Annu. Rev. Pharmacol. Toxicol..

[29] Itoh K., Chiba T., Takahashi S., Ishii T., Igarashi K., Katoh Y., Oyake T., Hayashi N., Satoh K., Hatayama I. (1997). An Nrf2/Small Maf Heterodimer Mediates the Induction of Phase II Detoxifying Enzyme Genes through Antioxidant Response Elements. Biochem. Biophys. Res. Commun..

[30] Venugopal R., Jaiswal A.K. (1996). Nrf1 and Nrf2 positively and c-Fos and Fra1 negatively regulate the human antioxidant response element-mediated expression of NAD(P)H:quinone oxidoreductase _1_ gene. Proc. Natl. Acad. Sci. USA.

[31] Larini A., Bianchi L., Bocci V. (2004). Effect of 4-hydroxynonenal on antioxidant capacity and apoptosis induction in Jurkat T cells. Free Radic. Res..

[32] Singhal S.S., Singh S.P., Singhal P., Horne D., Singhal J., Awasthi S. (2015). Antioxidant role of glutathione S-transferases: 4-Hydroxynonenal, a key molecule in stress-mediated signaling. Toxicol. Appl. Pharmacol..

[33] Nguyen T., Sherratt P.J., Pickett C.B. (2003). Regulatory Mechanisms Controlling Gene Expression Mediated by the Antioxidant Response Element. Annu. Rev. Pharmacol. Toxicol..

[34] Ma Q. (2008). Xenobiotic-Activated Receptors: From Transcription to Drug Metabolism to Disease. Chem. Res. Toxicol..

[35] Chirumbolo S., Valdenassi L., Simonetti V., Bertossi D., Ricevuti G., Franzini M., Pandolfi S. (2021). Insights on the mechanisms of action of ozone in the medical therapy against COVID-19. Int. Immunopharmacol..

[36] Kovac S., Angelova P.R., Holmström K.M., Zhang Y., Dinkova-Kostova A.T., Abramov A.Y. (2015). Nrf2 regulates ROS production by mitochondria and NADPH oxidase. Biochim. Biophys. Acta.

[37] Kasai S., Shimizu S., Tatara Y., Mimura J., Itoh K. (2020). Regulation of Nrf2 by Mitochondrial Reactive Oxygen Species in Physiology and Pathology. Biomolecules.

[38] Ryan D.G., Knatko E.V., Casey A.M., Hukelmann J.L., Naidu S.D., Brenes A.J., Ekkunagul T., Baker C., Higgins M., Tronci L. (2022). Nrf2 activation reprograms macrophage intermediary metabolism and suppresses the type I interferon response. iScience.

[39] Cattel F., Giordano S., Bertiond C., Lupia T., Corcione S., Scaldaferri M., Angelone L., De Rosa F.G. (2020). Ozone therapy in COVID-19: A narrative review. Virus Res..

[40] Izadi M., Cegolon L., Javanbakht M., Sarafzadeh A., Abolghasemi H., Alishiri G., Zhao S., Einollahi B., Kashaki M., Jonaidi-Jafari N. (2020). Ozone therapy for the treatment of COVID-19 pneumonia: A scoping review. Int. Immunopharmacol..

[41] Villani E., Ranaldi G., Franza L. (2020). Rationale for ozone-therapy as an adjuvant therapy in COVID-19: A narrative review. Med. Gas. Res..

[42] Shah M., Captain J., Vaidya V., Kulkarni A., Valsangkar K., Nair P.M., Ganu G. (2020). Safety and efficacy of ozone therapy in mild to moderate COVID-19 patients: A phase 1/11 randomized control trial (SEOT study). Int. Immunopharmacol..

[43] Hernández A., Viñals M., Pablos A., Vilás F., Papadakos P.J., Wijeysundera D.N., Bergese S.D., Vives M. (2020). Ozone therapy for patients with COVID-19 pneumonia: Preliminary report of a prospective case-control study. Int. Immunopharmacol..

[44] Çolak Ş., Yavuz B.G., Yavuz M., Özçelik B., Öner M., Özgültekin A., Şenbayrak S. (2021). Effectiveness of ozone therapy in addition to conventional treatment on mortality in patients with COVID-19. Int. J. Clin. Pract..

[45] Franzini M., Valdenassi L., Ricevuti G., Chirumbolo S., Depfenhart M., Bertossi D., Tirelli U. (2020). Oxygen-ozone (O2-O3) immunoceutical therapy for patients with COVID-19. Preliminary evidence reported. Int. Immunopharmacol..

[46] Cenci A., Macchia I., La Sorsa V., Sbarigia C., Di Donna V., Pietraforte D. (2022). Mechanisms of Action of Ozone Therapy in Emerging Viral Diseases: Immunomodulatory Effects and Therapeutic Advantages with Reference to SARS-CoV-2. Front. Microbiol..

[47] Hernández A., Viñals M., Isidoro T., Vilás F. (2020). Potential Role of Oxygen–Ozone Therapy in Treatment of COVID-19 Pneumonia. Am. J. Case Rep..

[48] Zheng Z., Dong M., Hu K. (2020). A preliminary evaluation on the efficacy of ozone therapy in the treatment of COVID-19. J. Med. Virol..

[49] Araimo F., Imperiale C., Tordiglione P., Ceccarelli G., Borrazzo C., Alessandri F., Santinelli L., Innocenti G.P., Pinacchio C., Mauro V. (2020). Ozone as adjuvant support in the treatment of COVID-19: A preliminary report of probiozovid trial. J. Med. Virol..

[50] Fernández-Cuadros M.E., Albaladejo-Florín M.J., Álava-Rabasa S., Gallego-Galiana J., Pérez-Cruz G.F., Usandizaga-Elio I., Pacios E., Torres-García D.E., Peña-Lora D., Casique-Bocanegra L. (2021). Compassionate Use of Rectal Ozone (O3) in Severe COVID-19 Pneumonia: A Case-Control Study. SN Compr. Clin. Med..

[51] Hendawy H.A., Mosallam W., Abuelnaga M.E., Sabry A.M. (2021). Old Treatment for a New Disease: Can Rectal Ozone Insufflation Be Used for COVID-19 Management? A Case Report. SN Compr. Clin. Med..

[52] Sharma A., Shah M., Sane H., Gokulchandran N., Paranjape A., Khubchandani P., Captain J., Shirke S., Kul-karni P. (2021). Intravenous ozonized saline therapy as prophylaxis for healthcare workers (HCWs) in a dedicated COVID-19 hospital in India –A retrospective study. Eur. Rev. Med. Pharm. Sci..

[53] Sozio E., De Monte A., Sermann G., Bassi F., Sacchet D., Sbrana F., Ripoli A., Curcio F., Fabris M., Marengo S. (2021). CORonavirus-19 mild to moderate pneumonia Management with blood Ozonization in patients with Respiratory failure (CORMOR) multicentric prospective randomized clinical trial. Int. Immunopharmacol..

[54] Sharma A., Shah M., Lakshmi S., Sane H., Captain J., Gokulchandran N., Khubchandani P., Pradeep M., Gote P., Tuppekar B. (2021). A pilot study for treatment of COVID-19 patients in moderate stage using intravenous administration of ozonized saline as an adjuvant treatment-registered clinical trial. Int. Immunopharmacol..

[55] Tascini C., Sermann G., Pagotto A., Sozio E., De Carlo C., Giacinta A., Sbrana F., Ripoli A., Castaldo N., Merelli M. (2020). Blood ozonization in patients with mild to moderate COVID-19 pneumonia: A single centre experience. Intern. Emerg. Med..

[56] Dengiz E., Özcan Ç., Güven Y.İ., Uçar S., Ener B.K., Sözen S., Yağcı B., Güzel İ.A., Yiğit B., Andaç A. (2022). Ozone Gas Applied through Nebulization as Adjuvant Treatment for Lung Respiratory Diseases Due to COVID-19 Infections: A Prospective Randomized Trial. Med. Gas. Res..

[57] Tirelli U., Franzini M., Valdenassi L., Pisconti S., Taibi R., Torrisi C., Pandolfi S., Chirumbolo S. (2021). Fatigue in Post-Acute Sequelae of SARS-CoV2 (PASC) Treated with Oxygen-Ozone Autohemotherapy-Preliminary Results on 100 Patients. Eur. Rev. Med. Pharm. Sci..

[58] Liu Q., Zhang D., Hu D., Zhou X., Zhou Y. (2018). The role of mitochondria in NLRP3 inflammasome activation. Mol. Immunol..

[59] Patergnani S., Bouhamida E., Leo S., Pinton P., Rimessi A. (2021). Mitochondrial Oxidative Stress and “Mito-Inflammation”: Actors in the Diseases. Biomedicines.

[60] Chen Y., Zhou Z., Min W. (2018). Mitochondria, Oxidative Stress and Innate Immunity. Front. Physiol..

[61] Bonaventura A., Vecchié A., Dagna L., Martinod K., Dixon D.L., Van Tassell B.W., Dentali F., Montecucco F., Massberg S., Levi M. (2021). Endothelial dysfunction and immunothrombosis as key pathogenic mechanisms in COVID-19. Nat. Rev. Immunol..

[62] Infantes E.C., Bautista J.T., Beltrán-Povea A., Cahuana G.M., Soria B., Nabil H., Bedoya F., Tejedo J.R. (2017). Regulation of mitochondrial function and endoplasmic reticulum stress by nitric oxide in pluripotent stem cells. World J. Stem Cells.

[63] Nisoli E., Falcone S., Tonello C., Cozzi V., Palomba L., Fiorani M., Pisconti A., Brunelli S., Cardile A., Francolini M. (2004). Mitochondrial biogenesis by NO yields functionally active mitochondria in mammals. Proc. Natl. Acad. Sci. USA.

[64] Valerio A., Nisoli E. (2015). Nitric oxide, interorganelle communication, and energy flow: A novel route to slow aging. Front. Cell Dev. Biol..

[65] Åkerström S., Mousavi-Jazi M., Klingström J., Leijon M., Lundkvist A., Mirazimi A. (2005). Nitric Oxide Inhibits the Replication Cycle of Severe Acute Respiratory Syndrome Coronavirus. J. Virol..

[66] Martínez M.C., Morell F.B., Raya A., Romá J., Aldasoro M., Vila J., Lluch S., Romero F.J. (1994). 4-Hydroxynonenal, a Lipid Peroxidation Product, Induces Relaxation of Human Cerebral Arteries. J. Cereb. Blood Flow Metab..

[67] Romero F.J., Romero M.J., Morell F.B., Martínez M., Medina P., Lluch S. (1997). 4-Hydroxynonenal-Induced Relaxation of Human Mesenteric Arteries. Free Radic. Biol. Med..

[68] Maulucci G., Daniel B., Cohen O., Avrahami Y., Sasson S. (2016). Hormetic and regulatory effects of lipid peroxidation mediators in pancreatic beta cells. Mol. Asp. Med..

[69] Chapple S.J., Cheng X., Mann G.E. (2013). Effects of 4-hydroxynonenal on vascular endothelial and smooth muscle cell redox signaling and function in health and disease. Redox Biol..

[70] Harry R.S., Hiatt L.A., Kimmel D.W., Carney C.K., Halfpenny K.C., Cliffel D.E., Wright D.W. (2012). Metabolic Impact of 4-Hydroxynonenal on Macrophage-Like RAW 264.7 Function and Activation. Chem. Res. Toxicol..

[71] Bocci V.A., Zanardi I., Travagli V. (2011). Ozone acting on human blood yields a hormetic dose-response relationship. J. Transl. Med..

[72] Shinriki N., Suzuki T., Takama K., Fukunaga K., Ohgiya S., Kubota K., Miura T. (1998). Susceptibilities of Plasma Antioxi-dants and Erythrocyte Constituents to Low Levels of Ozone. Haematologia (Budap).

[73] Gatbonton-Schwager T.N., Sadhukhan S., Zhang G.-F., Letterio J.J., Tochtrop G.P. (2014). Identification of a negative feedback loop in biological oxidant formation fegulated by 4-hydroxy-2-(E)-nonenal. Redox Biol..

[74] Bocci V., Aldinucci C., Mosci F., Carraro F., Valacchi G. (2007). Ozonation of Human Blood Induces a Remarkable Upregulation of Heme Oxygenase-1 and Heat Stress Protein-70. Mediat. Inflamm..

[75] Hull T.D., Boddu R., Guo L., Tisher C.C., Traylor A.M., Patel B., Joseph R., Prabhu S.D., Suliman H.B., Piantadosi C.A. (2016). Heme oxygenase-1 regulates mitochondrial quality control in the heart. JCI Insight.

[76] Korski K.I., Kubli D.A., Wang B.J., Khalafalla F.G., Monsanto M.M., Firouzi F., Echeagaray O.H., Kim T., Adamson R.M., Dembitsky W.P. (2019). Hypoxia Prevents Mitochondrial Dysfunction and Senescence in Human c-Kit+ Cardiac Progenitor Cells. Stem. Cells.

[77] Grieb P., Swiatkiewicz M., Prus K., Rejdak K. (2021). Hypoxia may be a determinative factor in COVID-19 progression. Curr. Res. Pharmacol. Drug Discov..

[78] Bansal S., Biswas G., Avadhani N.G. (2013). Mitochondria-targeted heme oxygenase-1 induces oxidative stress and mitochondrial dysfunction in macrophages, kidney fibroblasts and in chronic alcohol hepatotoxicity. Redox Biol..

[79] Shen H.-H., Wang C.-J., Zhang X.-Y., Sheng Y.-R., Yang S.-L., Zheng Z.-M., Shi J.-L., Qiu X.-M., Xie F., Li M.-Q. (2022). HIF1A-induced heme oxygenase 1 promotes the survival of decidual stromal cells against excess heme-mediated oxidative stress. Reproduction.

[80] Agani F.H., Puchowicz M., Chavez J.C., Pichiule P., LaManna J. (2002). Role of nitric oxide in the regulation of HIF-1α expression during hypoxia. Am. J. Physiol. Physiol..

[81] Deng L., Meng W., Li D., Qiu D., Wang S., Liu H. (2018). The Effect of Ozone on Hypoxia, Hemolysis and Morphological Change of Blood from Patients with Aortic Dissection (AD): A Preliminary in Vitro Experiment of Ozonated Autohemo-therapy for Treating AD. Am. J. Transl. Res..

[82] Tian M., Liu W., Li X., Zhao P., Shereen M.A., Zhu C., Huang S., Liu S., Yu X., Yue M. (2021). HIF-1α promotes SARS-CoV-2 infection and aggravates inflammatory responses to COVID-19. Signal Transduct. Target. Ther..

[83] Dunn L.L., Kong S.M., Tumanov S., Chen W., Cantley J., Ayer A., Maghzal G.J., Midwinter R.G., Chan K.H., Ng M.K. (2020). Hmox1 (Heme Oxygenase-1) Protects Against Ischemia-Mediated Injury via Stabilization of HIF-1α (Hypoxia-Inducible Factor-1α). Arter. Thromb. Vasc. Biol..

[84] Papandreou I., Cairns R.A., Fontana L., Lim A.L., Denko N.C. (2006). HIF-1 mediates adaptation to hypoxia by actively downregulating mitochondrial oxygen consumption. Cell Metab..

[85] Pagé E.L., Chan D.A., Giaccia A.J., Levine M., Richard D.E. (2008). Hypoxia-inducible Factor-1α Stabilization in Nonhypoxic Conditions: Role of Oxidation and Intracellular Ascorbate Depletion. Mol. Biol. Cell.

[86] Jay R.R., Howard R. (2020). A Plausible “Penny” Costing Effective Treatment for Corona Virus-Ozone Therapy. J. Infect. Dis. Epidemiol..

[87] Fernández-Cuadros M.E., Albaladejo-Florín M.J., Peña-Lora D., Álava-Rabasa S., Pérez-Moro O.S. (2020). Ozone (O3) and SARS-CoV-2: Physiological Bases and Their Therapeutic Possibilities According to COVID-19 Evolutionary Stage. SN Compr. Clin. Med..

[88] Manjunath S.N., Sakar M., Katapadi M., Balakrishna R.G. (2020). Recent case studies on the use of ozone to combat coronavirus: Problems and perspectives. Environ. Technol. Innov..

[89] Yilmaz N., Eren E., Oz C. (2021). COVID-19 and Ozone. Cyprus. J. Med. Sci..

[90] Yousefi B., Banihashemian S.Z., Feyzabadi Z.K., Hasanpour S., Kokhaei P., Abdolshahi A., Emadi A., Eslami M. (2022). Potential Therapeutic Effect of Oxygen-Ozone in Controlling of COVID-19 Disease. Med. Gas. Res..

[91] Gu Y.Q. (2022). Suggestion for a Long-Term Effective Disinfection to Eliminate the COVID-19 Epidemic. BAOJ Microbiol..

[92] Bocci V., Valacchi G. (2015). Nrf2 activation as target to implement therapeutic treatments. Front. Chem..

[93] Falero-Perez J., Song Y.-S., Sorenson C.M., Sheibani N. (2018). CYP1B1: A key regulator of redox homeostasis. Trends Cell Mol. Biol..

[94] Wang G., Xiao B., Deng J., Gong L., Li Y., Li J., Zhong Y. (2022). The Role of Cytochrome P450 Enzymes in COVID-19 Pathogenesis and Therapy. Front. Pharmacol..

[95] Afaq F., Abu Zaid M., Pelle E., Khan N., Syed D.N., Matsui M.S., Maes D., Mukhtar H. (2009). Aryl Hydrocarbon Receptor Is an Ozone Sensor in Human Skin. J. Investig. Dermatol..

[96] Jacob A., Hartz A.M., Potin S., Coumoul X., Yousif S., Scherrmann J.-M., Bauer B., Declèves X. (2011). Aryl hydrocarbon receptor-dependent upregulation of Cyp1b1 by TCDD and diesel exhaust particles in rat brain microvessels. Fluids Barriers CNS.

[97] Chirumbolo S. (2010). The Role of Quercetin, Flavonols and Flavones in Modulating Inflammatory Cell Function. Inflamm. Allergy-Drug Targets.

[98] Kvarantan A., Balta V., Zarkovic N., Horvat T., Vukovic T., Zarkovic K., Kalogjera L. (2021). Association between aryl hydrocarbon receptor and 4-hydroxynonenal in oxidative stress-mediated chronic rhinosinusitis with nasal polyps. Eur. J. Inflamm..

[99] Larigot L., Benoit L., Koual M., Tomkiewicz C., Barouki R., Coumoul X. (2022). Aryl Hydrocarbon Receptor and Its Diverse Ligands and Functions: An Exposome Receptor. Annu. Rev. Pharmacol. Toxicol..

[100] Hwang H.J., Dornbos P., Steidemann M., Dunivin T.K., Rizzo M., LaPres J.J. (2016). Mitochondrial-targeted aryl hydrocarbon receptor and the impact of 2,3,7,8-tetrachlorodibenzo-p-dioxin on cellular respiration and the mitochondrial proteome. Toxicol. Appl. Pharmacol..

[101] Dwier M., Michalek R., Saloupis P., McDonnell D., Malek G. (2011). Oxidized Lipids Activate Aryl Hydrocarbon Receptor (AhR) and Differentially Regulate Metabolic Pathways in Retinal Pigment Epithelial Cells (RPE). Invest. Ophtalmol. Vis. Sci..

[102] Mao K., Chen S., Chen M., Ma Y., Wang Y., Huang B., He Z., Zeng Y., Hu Y., Sun S. (2013). Nitric oxide suppresses NLRP3 inflammasome activation and protects against LPS-induced septic shock. Cell Res..

[103] Laskin D.L., Sunil V., Guo Y., E Heck D., Laskin J.D. (1998). Increased nitric oxide synthase in the lung after ozone inhalation is associated with activation of NF-kappa B. Environ. Health Perspect..

[104] Hogg N., Kalyanaraman B. (1999). Nitric oxide and lipid peroxidation. Biochim. Biophys. Acta.

[105] Anderson E.J., A Katunga L., Willis M.S. (2011). Mitochondria as a source and target of lipid peroxidation products in healthy and diseased heart. Clin. Exp. Pharmacol. Physiol..

[106] Ceban F., Ling S., Lui L.M., Lee Y., Gill H., Teopiz K.M., Rodrigues N.B., Subramaniapillai M., Di Vincenzo J.D., Cao B. (2021). Fatigue and cognitive impairment in Post-COVID-19 Syndrome: A systematic review and meta-analysis. Brain Behav. Immun..

[107] Sudre C.H., Murray B., Varsavsky T., Graham M.S., Penfold R.S., Bowyer R.C., Pujol J.C., Klaser K., Antonelli M., Canas L.S. (2021). Attributes and predictors of long COVID. Nat. Med..

[108] Merad M., Blish C.A., Sallusto F., Iwasaki A. (2022). The immunology and immunopathology of COVID-19. Science.

[109] Andrade B.S., Siqueira S., Soares W.D.A., Rangel F.D.S., Santos N., Freitas A.D.S., da Silveira P.R., Tiwari S., Alzahrani K., Góes-Neto A. (2021). Long-COVID and Post-COVID Health Complications: An Up-to-Date Review on Clinical Conditions and Their Possible Molecular Mechanisms. Viruses.

[110] Evans R.A., Leavy O.C., Richardson M., Elneima O., McCauley H.J.C., Shikotra A., Singapuri A., Sereno M., Saunders R.M., Harris V.C. (2022). Clinical characteristics with inflammation profiling of long COVID and association with 1-year recovery following hospitalisation in the UK: A prospective observational study. Lancet Respir. Med..

[111] Weinstock L.B., Brook J.B., Walters A.S., Goris A., Afrin L.B., Molderings G.J. (2021). Mast cell activation symptoms are prevalent in Long-COVID. Int. J. Infect. Dis..

[112] Kappelmann N., Dantzer R., Khandaker G.M. (2021). Interleukin-6 as potential mediator of long-term neuropsychiatric symptoms of COVID-19. Psychoneuroendocrinology.

[113] Phetsouphanh C., Darley D.R., Wilson D.B., Howe A., Munier C.M.L., Patel S.K., Juno J.A., Burrell L.M., Kent S.J., Dore G.J. (2022). Immunological dysfunction persists for 8 months following initial mild-to-moderate SARS-CoV-2 infection. Nat. Immunol..

[114] Doykov I., Hällqvist J., Gilmour K.C., Grandjean L., Mills K., Heywood W.E. (2021). ‘The long tail of Covid-19’-The detection of a prolonged inflammatory response after a SARS-CoV-2 infection in asymptomatic and mildly affected patients. F1000Research.

[115] Chang J.D.S., Lu H.-S., Chang Y.-F., Wang D. (2004). Ameliorative effect of ozone on cytokine production in mice injected with human rheumatoid arthritis synovial fibroblast cells. Rheumatol. Int..

[116] Martínez-Sánchez G., Schwartz A., Di Donna V. (2020). Potential Cytoprotective Activity of Ozone Therapy in SARS-CoV-2/COVID-19. Antioxidants.

[117] Varesi A., Chirumbolo S., Ricevuti G. (2021). Oxygen–ozone treatment and COVID-19: Antioxidants targeting endothelia lead the scenery. Intern. Emerg. Med..

[118] Wei A., Feng H., Jia X.-M., Tang H., Liao Y.-Y., Li B.-R. (2018). Ozone therapy ameliorates inflammation and endometrial injury in rats with pelvic inflammatory disease. Biomed. Pharmacother..

[119] Tahmasebi S., Qasim M.T., Krivenkova M.V., Zekiy A.O., Thangavelu L., Aravindhan S., Izadi M., Jadidi-Niaragh F., Ghaebi M., Aslani S. (2021). The effects of oxygen–ozone therapy on regulatory T-cell responses in multiple sclerosis patients. Cell Biol. Int..

[120] Díaz-Resendiz K.J.G., Benitez-Trinidad A.B., Covantes-Rosales C.E., Toledo-Ibarra G.A., Ortiz-Lazareno P.C., Girón-Pérez D.A., Bueno-Durán A.Y., Pérez-Díaz D.A., Barcelos-García R.G., Girón-Pérez M.I. (2022). Loss of mitochondrial membrane potential (Δ *Ψ* _m_ ) in leucocytes as post-COVID-19 sequelae. J. Leukoc. Biol..

[121] Galam L., Failla A., Soundararajan R., Lockey R.F., Kolliputi N. (2015). 4-Hydroxynonenal regulates mitochondrial function in human small airway epithelial cells. Oncotarget.

[122] Rai P., Janardhan K.S., Meacham J., Madenspacher J.H., Lin W.-C., Karmaus P.W.F., Martinez J., Li Q.-Z., Yan M., Zeng J. (2021). IRGM1 links mitochondrial quality control to autoimmunity. Nat. Immunol..

[123] Paul B.D., Lemle M.D., Komaroff A.L., Snyder S.H. (2021). Redox imbalance links COVID-19 and myalgic encephalomyelitis/chronic fatigue syndrome. Proc. Natl. Acad. Sci. USA.

[124] Vollbracht C., Kraft K. (2022). Oxidative Stress and Hyper-Inflammation as Major Drivers of Severe COVID-19 and Long COVID: Implications for the Benefit of High-Dose Intravenous Vitamin C. Front. Pharmacol..

[125] Brodin P., Casari G., Townsend L., O’Farrelly C., Tancevski I., Löffler-Ragg J., Mogensen T.H., Casanova J.L., Abel L., Aiuti A. (2022). Studying severe long COVID to understand post-infectious disorders beyond COVID-19. Nat. Med..

[126] Clavo B., Rodríguez-Esparragón F., Rodríguez-Abreu D., Martínez-Sánchez G., Llontop P., Aguiar-Bujanda D., Fernández-Pérez L., Santana-Rodríguez N. (2019). Modulation of Oxidative Stress by Ozone Therapy in the Prevention and Treatment of Chemotherapy-Induced Toxicity: Review and Prospects. Antioxidants.

[127] Galiè M., Costanzo M., Nodari A., Boschi F., Calderan L., Mannucci S., Covi V., Tabaracci G., Malatesta M. (2018). Mild ozonisation activates antioxidant cell response by the Keap1/Nrf2 dependent pathway. Free. Radic. Biol. Med..

[128] Mohamed M.S., Johansson A., Jonsson J., Schiöth H.B. (2022). Dissecting the Molecular Mechanisms Surrounding Post-COVID-19 Syndrome and Neurological Features. Int. J. Mol. Sci..

[129] Ercegovac M., Asanin M., Savic-Radojevic A., Ranin J., Matic M., Djukic T., Coric V., Jerotic D., Todorovic N., Milosevic I. (2022). Antioxidant Genetic Profile Modifies Probability of Developing Neurological Sequelae in Long-COVID. Antioxidants.

[130] Jarrott B., Head R., Pringle K.G., Lumbers E.R., Martin J.H. (2022). “LONG COVID”—A hypothesis for understanding the biological basis and pharmacological treatment strategy. Pharmacol. Res. Perspect..

[131] Crook H., Raza S., Nowell J., Young M., Edison P. (2021). Long covid—mechanisms, risk factors, and management. BMJ.

[132] Di Girolamo F.G., Fiotti N., Sisto U.G., Nunnari A., Colla S., Mearelli F., Vinci P., Schincariol P., Biolo G. (2022). Skeletal Muscle in Hypoxia and Inflammation: Insights on the COVID-19 Pandemic. Front. Nutr..

[133] Karaarslan F., Güneri F.D., Kardeş S. (2021). Long COVID: Rheumatologic/musculoskeletal symptoms in hospitalized COVID-19 survivors at 3 and 6 months. Clin. Rheumatol..

[134] Khan S.A., Seyam O., Smith N.L., Reid I., Gandhi J., Jiang W. (2018). Clinical utility of ozone therapy for musculoskeletal disorders. Med. Gas. Res..

[135] De Sire A., Agostini F., Lippi L., Mangone M., Marchese S., Cisari C., Bernetti A., Invernizzi M. (2021). Oxygen–Ozone Therapy in the Rehabilitation Field: State of the Art on Mechanisms of Action, Safety and Effectiveness in Patients with Musculoskeletal Disorders. Biomolecules.

[136] De Sire A., Marotta N., Ferrillo M., Agostini F., Sconza C., Lippi L., Respizzi S., Giudice A., Invernizzi M., Ammendolia A. (2022). Oxygen-Ozone Therapy for Reducing Pro-Inflammatory Cytokines Serum Levels in Musculoskeletal and Temporomandibular Disorders: A Comprehensive Review. Int. J. Mol. Sci..

[137] Akkawi I. (2020). Ozone therapy for musculoskeletal disorders Current concepts. Acta Biomed..

[138] Tirelli U., Franzini M., Valdenassi L., Pandolfi S., Berretta M., Ricevuti G., Chirumbolo S. (2021). Patients with Myalgic Encephalomyelitis/Chronic Fatigue Syndrome (ME/CFS) Greatly Improved Fatigue Symptoms When Treated with Oxygen-Ozone Autohemotherapy. J. Clin. Med..

[139] Tirelli U., Cirrito C., Pavanello M., Piasentin C., Lleshi A., Taibi R. (2019). Ozone therapy in 65 patients with fibromyalgia: An effective therapy. Eur. Rev. Med. Pharmacol. Sci..

[140] Li Y., Feng X., Ren H., Huang H., Wang Y., Yu S. (2021). Low-Dose Ozone Therapy Improves Sleep Quality in Patients with Insomnia and Coronary Heart Disease by Elevating Serum BDNF and GABA. Bull. Exp. Biol. Med..

[141] Moghimi N., Di Napoli M., Biller J., Siegler J.E., Shekhar R., McCullough L.D., Harkins M.S., Hong E., Alaouieh D.A., Mansueto G. (2021). The Neurological Manifestations of Post-Acute Sequelae of SARS-CoV-2 infection. Curr. Neurol. Neurosci. Rep..

[142] Mondelli V., Pariante C.M. (2021). What can neuroimmunology teach us about the symptoms of long-COVID?. Oxf. Open Immunol..

[143] Scassellati C., Ciani M., Galoforo A.C., Zanardini R., Bonvicini C., Geroldi C. (2020). Molecular mechanisms in cognitive frailty: Potential therapeutic targets for oxygen-ozone treatment. Mech. Ageing Dev..

[144] Scassellati C., Galoforo A.C., Esposito C., Ciani M., Ricevuti G., Bonvicini C. (2021). Promising Intervention Approaches to Potentially Resolve Neuroinflammation And Steroid Hormones Alterations in Alzheimer’s Disease and Its Neuropsychiatric Symptoms. Aging Dis..

[145] Clavo B., Santana-Rodríguez N., Gutierrez D., Lopez J.C., Suarez G., Lopez L., Robaina F., Bocci V. (2013). Long-Term Improvement in Refractory Headache Following Ozone Therapy. J. Altern. Complement. Med..

[146] Haggiag S., Prosperini L., Stasolla A., Gerace C., Tortorella C., Gasperini C. (2021). Ozone-induced encephalopathy: A novel iatrogenic entity. Eur. J. Neurol..

[147] Rangel K., Cabral F.O., Lechuga G.C., Carvalho J.P.R.S., Villas-Bôas M.H.S., Midlej V., De-Simone S.G. (2022). Potent Activity of a High Concentration of Chemical Ozone against Antibiotic-Resistant Bacteria. Molecules.

[148] Roede J.R., Jones D.P. (2010). Reactive species and mitochondrial dysfunction: Mechanistic significance of 4-hydroxynonenal. Environ. Mol. Mutagen..

[149] Dodson M., Wani W.Y., Redmann M., Benavides G.A., Johnson M.S., Ouyang X., Cofield S.S., Mitra K., Darley-Usmar V., Zhang J. (2017). Regulation of autophagy, mitochondrial dynamics, and cellular bioenergetics by 4-hydroxynonenal in primary neurons. Autophagy.

